# COVID-19 Vaccination in Pregnancy and Lactation: Current Research and Gaps in Understanding

**DOI:** 10.3389/fcimb.2021.735394

**Published:** 2021-09-16

**Authors:** Lydia L. Shook, Parisa N. Fallah, Jason N. Silberman, Andrea G. Edlow

**Affiliations:** ^1^Department of Obstetrics and Gynecology, Massachusetts General Hospital, Harvard Medical School, Boston, MA, United States; ^2^Vincent Center for Reproductive Biology, Massachusetts General Hospital, Boston, MA, United States

**Keywords:** COVID-19, pregnancy, lactation, maternal immunization, vaccination, SARS-CoV-2, mRNA vaccine

## Abstract

The COVID-19 pandemic has demonstrated the urgent need to develop vaccine strategies optimized for pregnant people and their newborns, as both populations are at risk of developing severe disease. Although not included in COVID-19 vaccine development trials, pregnant people have had access to these vaccines since their initial release in the US and abroad. The rapid development and distribution of novel COVID-19 vaccines to people at risk, including those who are pregnant and lactating, presents an unprecedented opportunity to further our understanding of vaccine-induced immunity in these populations. In this review, we aim to summarize the literature to date on COVID-19 vaccination in pregnancy and lactation and highlight opportunities for investigation that may inform future maternal vaccine development and implementation strategies.

## Introduction

Vaccination against infectious pathogens is one of the most impactful public health interventions, reducing global morbidity and mortality related to infection (Woodworth et al., 2020; CDC, a, 2021). In the US alone, nearly 99,000 pregnant people have been infected with COVID-19, resulting in 109 maternal deaths to date (CDC, a, 2021). The COVID-19 pandemic has demonstrated the urgent need to develop vaccine strategies optimized for pregnant people and their newborns, as both populations are at risk of developing severe disease (Woodworth et al., 2020; Zambrano et al., 2020).

To date, two COVID-19 mRNA vaccines – BNT162b2 (Pfizer/BioNTech) and mRNA-1273 (Moderna) – and one monovalent Ad26-vector vaccine (Janssen/Johnson & Johnson) have been granted Emergency Use Authorization (EUA) by the FDA for administration to prevent COVID-19 in the US. Although not included in COVID-19 vaccine development trials, pregnant people have had access to these vaccines since their initial release in the US, and more recent data supporting the safety of COVID-19 vaccines in pregnancy (Shimabukuro et al., 2021) have led to broadening support for vaccinating pregnant and lactating individuals (ACOG, 2021 Immunization, Infectious Disease and Public Health Preparedness ExpertWorking Group; CDC, b, 2021). With increasing numbers of pregnant people receiving the vaccine during all trimesters of pregnancy and during lactation, several key questions have arisen, including: what is the safety profile of mRNA vaccines in pregnancy and lactation? Which vaccines induce the most robust maternal immune response? Does the efficiency of transplacental and breastmilk antibody transfer differ by timing of administration or vaccine platform? What factors govern efficiency of placental and breastmilk transfer? Does the transfer of humoral immunity from mother to baby confer long-lasting protection?

**Figure 1:**
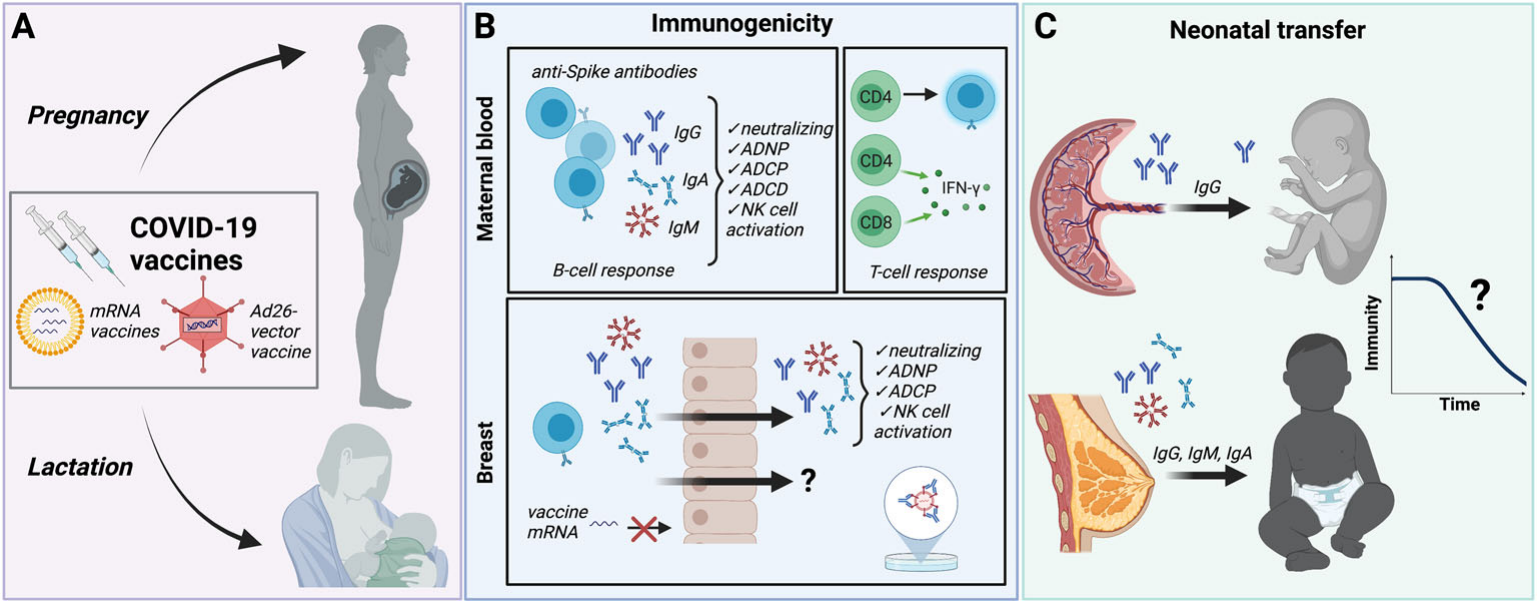
Summary of immune protection from COVID-19 vaccines in pregnancy and lactation. **(A)** Although excluded from initial vaccine trials, pregnant and lactating individuals have been eligible to receive Pfizer/BioNTech and Moderna mRNA vaccines and the Janssen/Johnson&Johnson Ad26-vector vaccine. Safety and reactogenicity profiles are similar to non-pregnant, non-lactating individuals. Immunization with the two-dose protocol for mRNA vaccines results in comparable IgG, IgA and IgM titers in fully-vaccinated pregnant and lactating individuals compared to non-pregnant controls. The longevity of immunity derived during pregnancy and the ability to confer protection against variants has not been directly studied in these populations. **(B)** COVID-19 vaccines generate anti-Spike antibodies in pregnant and lactating individuals with similar immunogenicity compared with non-pregnant controls. Vaccine-induced anti-Spike antibodies demonstrate neutralizing capacity, antibody-dependent neutrophil phagocytosis (ADNP), antibody-dependent cellular phagocytosis (ADCP), and antibody-dependent complement deposition (ADCD), and NK cell activation. Spike-specific CD4+ and CD8+ T-cell activity is similar to that observed in non-pregnant individuals. Anti-Spike IgG and IgA with binding, neutralizing and functional activity are also detectable in breastmilk. Whether breastmilk contains vaccine-induced cellular or other protective immune factors is not yet known. No vaccine mRNA has been detected in breastmilk immediately following vaccination. **(C)** Neutralizing anti-Spike IgG is transplacentally transferred from mother to fetus. Vaccine timing and maternal antibody titers impact cord titers. IgG, IgM and IgA are transferred through breastmilk. Neither the amount of maternally-derived antibodies required to confer neonatal protection from COVID-19 infection, nor the duration of this protection, is known. Created with BioRender.com. ADNP, antibody-dependent neutrophil phagocytosis; ADCP, antibody-dependent cellular phagocytosis; ADCD, antibody-dependent complement deposition.

The COVID-19 pandemic and the rapid development of novel vaccines to combat it present an unprecedented opportunity to decode the rules of vaccine-induced immunity in pregnant and lactating individuals. In this review, we aim to review the literature to date on COVID-19 vaccination in pregnancy and lactation and highlight opportunities for future investigation. **Figure 1** illustrates the key findings presented in this review as well as gaps in understanding. Knowledge gained through investigation of COVID-19 vaccines in pregnant and lactating people has the potential to lead to a deeper understanding of vaccine-induced immunity in pregnant individuals and their newborns, and to inform future vaccine development and implementation strategies.

## Safety of COVID-19 Vaccines in Reproduction

Because pregnant and lactating people were not included in initial vaccine trials, data on the vaccine safety and efficacy in these populations has been limited (Bianchi et al., 2021), and guidance from public health officials has been vague and at times conflicting (Adhikari and Spong, 2021). Available data on vaccine safety in pregnancy from Development and Reproductive Toxicity (DART) studies were overall reassuring, although limited in scope. A report submitted to the European Medicines Agency demonstrated that female rats injected with 4 human doses of Pfizer/BioNTech vaccine before and during gestation had no vaccine-related effects on female fertility, pregnancy or embryo-fetal or postnatal development (Pfizer/BioNTech, 2020). Similarly, a DART study of Moderna’s vaccine reviewed by the FDA found no adverse effects on reproduction or development when administered to rats at human doses (ModernaTX, Inc, 2020), and the FDA-reviewed Janssen COVID-19 vaccine DART study found no adverse effect on fertility, embryo-fetal, or postnatal development when twice the human dose was injected in female rabbits 7 days before mating and at gestational days 6 and 20 (early and late gestation) [U.S. Food and Drug Administration (FDA), 2021].

Experience from other vaccines utilizing the mRNA and adenovirus-vector platforms has provided some reassurance that neither technology carries specific reproductive safety concerns. Although pregnant people were excluded from Phase 1 trials of mRNA vaccine platforms against other pathogens such as influenza, Zika HIV and rabies viruses (Alberer et al., 2017; Feldman et al., 2019; Maruggi et al., 2019), animal studies of the mRNA vaccine against Zika virus demonstrated that vaccination of non-pregnant mice prior to gestation protected against transplacental transmission without any vaccine-associated reproductive safety events (Richner et al., 2017). In an analysis of 1,522 pregnancy cases from ongoing Ad26-vectored vaccine trials for Ebola vaccine, in which pregnant people are eligible to receive the vaccine, no pregnancy-related safety concerns were identified (JANSSEN BIOTECH, INC, 2021).

Despite the exclusion of pregnant and lactating people from COVID-19 vaccine trials, the American College of Obstetricians and Gynecologists as well as the Society for Maternal-Fetal Medicine have consistently voiced that the COVID-19 vaccine should be available to pregnant and lactating individuals, and both professional societies as well as the CDC now recommend vaccination in these populations (ACOG, 2021Immunization, Infectious Disease and Public Health Preparedness Expert Working Group; CDC, b, 2021). Inclusion of pregnant and lactating individuals choosing to receive the COVID-19 vaccine in observational studies and vaccine safety monitoring programs has been essential to generating additional safety data in these populations. A study of 84 pregnant, 31 lactating, and 16 non-pregnant women receiving either the Pfizer/BioNTech or Moderna vaccine also demonstrated no major adverse events and similar reactogenicity profiles between groups (Gray et al., 2021). In a study of 84 breastfeeding people who were vaccinated with the Pfizer/BioNTech vaccine in Israel, no mother or infant experienced any serious adverse event during the study period (Perl et al., 2021), and a recent study of 7 lactating individuals who received either the Pfizer/BioNTech or Moderna vaccine did not detect any vaccine-associated mRNA in breastmilk collected between 4 and 48 hours after vaccination (Golan et al., 2021).

Citing as rationale the fact that mRNA vaccines induce an immune response through toll-like receptor 3 (TLR3) activation, and TLR3 activation has been linked to adverse placentally-mediated pregnancy outcomes in rodent models such as decidual arteriopathy, growth restriction, preterm delivery, and fetal loss (Zhang et al., 2007; Koga et al., 2009; Thaxton et al., 2013; Pardi et al., 2018; Baines et al., 2020), another study of 84 pregnant people receiving the COVID-19 mRNA vaccines in pregnancy examined the placenta for lesions detectable on H&E histopathology (Shanes et al., 2021). These investigators found no increased incidence of decidual arteriopathy, fetal vascular malperfusion, low-grade chronic villitis, or chronic histiocytic intervillositis in the 84 people receiving COVID-19 vaccines in pregnancy compared with 116 unvaccinated pregnant individuals in the control group. No studies have yet examined the placental immune response to COVID-19 vaccines at a molecular and cellular level, and such studies would be able to detect subtler impacts of the COVID-19 vaccines on placental inflammation and function.

The CDC V-safe COVID-19 Pregnancy Registry team has recently published preliminary findings on the safety of mRNA COVID-19 vaccines in pregnant persons (Shimabukuro et al., 2021). A total of 35,691 pregnant individuals were identified from the “v-safe after vaccination health checker” surveillance system, the v-safe pregnancy registry, and the Vaccine Adverse Event Reporting System (VAERS). Of the 827 completed pregnancies, the incidences of adverse pregnancy and neonatal outcomes including pregnancy loss (13.9%), preterm birth (9.4%), and small for gestational age (3.2%) approximates pre-pandemic rates, and no obvious safety signals among pregnant individuals were identified (Shimabukuro et al., 2021). Official guidance from the CDC states that because no safety concerns were identified for pregnant people who were vaccinated or for their babies, pregnant people can receive the COVID-19 vaccine, and that approval from a care provider is not required (CDC, c, 2021). Due to reports of rare cases of blood clots with thrombocytopenia occurring in women younger than 50 years old receiving the J&J vaccine, the CDC does state that pregnant people should be aware of these risks, and of the availability of other vaccines for which this risk has not been observed (CDC, c, 2021).

Long term vaccine safety data in pregnant people and their offspring are understandably lacking at this time, yet critically needed for counseling regarding not only the safety of the COVID-19 vaccines specifically but for mRNA vaccine platforms in general, as public perception regarding the safety of vaccines given in pregnancy hinges on providing high-quality information on neonatal and childhood outcomes, including birth defects and childhood developmental disorders. Given the longer time horizon necessary to collect these data, they are unlikely to inform current vaccine practice, but do have the opportunity to impact future mRNA and adenovirus-vector vaccine development efforts and public health campaigns.

## Immunogenicity and Implications of Host-Virus Interactions in Pregnancy

Pregnancy involves complex immunological changes, including modulation of the immune system to tolerate the fetal semi-allograft (Jennewein et al., 2017; Than et al., 2019). Along with physiological and hormonal changes, these immunological adaptations contribute to the observed increased vulnerability of pregnant people to complications from viral respiratory infections, including H1N1 influenza and SARS-CoV-2 (Raj et al., 2014; Alberca et al., 2020). Evidence suggests that vaccine-induced immune responses may also differ in pregnancy (Saeed et al., 2020), with some studies demonstrating a less robust increase in post-vaccine titers in pregnant compared to non-pregnant women in response to H1N1 influenza and Tdap vaccines (Fortner et al., 2018; Schlaudecker et al., 2018).

Both COVID-19 mRNA vaccines (Pfizer/BioNTech, Moderna) and the Ad26-vector vaccine (Janssen) are highly immunogenic in non-pregnant populations (Walsh et al., 2020; Chu et al., 2021; Stephenson et al., 2021), generating robust post-vaccination anti-SARS-CoV-2-specific antibody titers in most study participants (Walsh et al., 2020). Emerging data suggest that pregnant people mount a serological response to COVID-19 mRNA vaccines that is comparable to reproductive aged, non-pregnant controls (Beharier et al., 2021; Collier et al., 2021; Gray et al., 2021; Prabhu et al., 2021). In a cohort of 84 pregnant, 31 lactating, and 16 non-pregnant reproductive-aged women, mRNA vaccine-induced titers of SARS-CoV-2 Spike and receptor-binding domain (RBD) IgG, IgA and IgM were equivalent between all groups (Gray et al., 2021). Vaccine-induced antibody titers did not differ by trimester of vaccination, and the second vaccine dose, i.e. “boost” dose, increased SARS-CoV-2 specific IgG in maternal blood and breastmilk. A systems serology analysis of the humoral immune response in the same participants demonstrated delayed kinetics of FcR-binding and antibody effector functions in both pregnant and lactating people compared to non-pregnant women, highlighting the importance of strict adherence to the prime/boost schedule to achieve full immunity in this vulnerable population (Atyeo et al., 2021a). Both studies also suggested differences in the humoral immune response to the Pfizer/BioNTech *versus* Moderna vaccine, including a more robust IgA immune response and higher antibody titers and functions induced by the Moderna compared to the Pfizer/BioNTech vaccine (Atyeo et al., 2021a; Gray et al., 2021). Whether there are clinical implications of these subtle differences between the Moderna and Pfizer/BioNTech vaccine is unclear, but the robust humoral immune response to both COVID-19 mRNA vaccines suggests both are likely to be highly efficacious in pregnancy. In another cohort of 122 pregnant people who had received at least one dose of mRNA vaccine prior to delivery, all participants demonstrated evidence of SARS-CoV-2-specific IgG antibody response by 4 weeks after first vaccine dose (Prabhu et al., 2021). Taken together, these data suggest that pregnant and lactating people can mount a serological response to the vaccine comparable to non-pregnant counterparts, with a similar IgG response to the vaccine boost as in non-pregnant controls.

Whether COVID-19 vaccines generate an equivalent or greater anti-SARS-CoV-2 antibody response against the SARS-CoV-2 virus compared to those antibodies generated by natural SARS-CoV-2 infection in pregnancy warrants additional investigation. In a study of 84 pregnant and 31 lactating participants, vaccine-induced anti-SARS-CoV-2-specific antibody titers were significantly higher in all participants than those induced by natural SARS-CoV-2 infection during pregnancy. In this cohort, participants with natural infection were symptomatic and known to be infected 4 to 12 weeks prior to titer quantification, so that timing of antibody quantification was comparable for the pregnant vaccinees and naturally-infected pregnant people (Gray et al., 2021). This significant increase in antibody response after COVID vaccination compared to natural infection in pregnancy was also observed in a prospective cohort of 103 women, which included 30 pregnant participants who received either mRNA vaccine during pregnancy and 28 participants infected with SARS-CoV-2 in pregnancy (Collier et al., 2021).

Not only antibody titer, but antibody function is a key consideration in evaluating vaccine-induced antibody protection for both mother and newborn. Two studies have evaluated antibody function in pregnant and lactating compared to non-pregnant women receiving the COVID-19 mRNA vaccines (Atyeo et al., 2021a; Collier et al., 2021). Both have demonstrated similar spike-specific antibody-dependent neutrophil phagocytosis (ADNP), antibody-dependent complement deposition (ADCD), and antibody-dependent cellular phagocytosis (ADCP) in fully vaccinated pregnant and lactating compared to non-pregnant individuals. In comparing spike-specific antibody functional profiles after the “prime” and “boost” doses in pregnant people to lactating/non-pregnant controls, Atyeo and colleagues identified initial differences in assay responses suggestive of impaired antibody functionality following the “prime” dose in pregnant individuals, which improved following the “boost” dose (Atyeo et al., 2021a). In their observational study including 30 pregnant, 16 lactating, and 57 non-pregnant individuals, Collier and colleagues also assessed cellular immune responses to the mRNA COVID-19 vaccines, quantifying the percent of spike-specific IFN-γ production by CD4 T cells, CD4 central memory T cells, CD8 T cells, and CD8 central memory T cells. This study reported comparable cellular immune responses to the COVID-19 vaccines in pregnant, lactating, and non-pregnant women, and demonstrated that the mRNA vaccines generated humoral and cellular responses against SARS-CoV-2 variants of concern B.1.1.7 and B.1.351. Taken together, these data support robust humoral and cellular immune response to COVID-19 mRNA vaccines in individuals vaccinated during pregnancy, although given the delayed antibody kinetics observed in pregnant compared to non-pregnant individuals, adherence to recommended prime/boost mRNA vaccine schedules may be especially critical in pregnancy to achieve immunity comparable to that in non-pregnant populations (Atyeo et al., 2021a). To date, no group has yet reported on the antibody response to the Janssen vaccine specifically in pregnant and lactating individuals.

Recent data investigating the vaccine-induced cellular and serological immune response in individuals previously infected with SARS-CoV-2 points to a robust response to the first vaccine dose in both circulating antibodies and antigen-specific memory B cells (Goel et al., 2021; Krammer et al., 2021). A study of previously SARS-CoV-2-infected *versus* uninfected vaccinated healthcare workers found evidence that after the administration of a single dose of vaccine, the humoral response in individuals with a history of SARS-CoV-2 infection is greater than the response in previously uninfected participants who have received a second dose (Anichini et al., 2021). Given the relatively weak serological response to natural infection in pregnant people (Edlow et al., 2020; Gray et al., 2021), investigating both the serological and cellular response to COVID-19 vaccines in people previously infected with SARS-CoV-2 during pregnancy will be critical to development of additional recommendations regarding vaccine dosing in individuals who were infected during pregnancy.

## Protection at Birth: Maternal-Fetal Transplacental Antibody Transfer Following Vaccination

Compared to pediatric and adult populations, vaccine administration in newborns has been less effective at reducing infection-related deaths (Amenyogbe et al., 2015; Kollmann et al., 2017). Compromised vaccine-induced immunity in infants has been attributed to the potentially tolerogenic nature of the neonatal immune system (Yu et al., 2018), the less functional nature of newborn immune cells (Lee and Lin, 2013; Yu et al., 2018), and dampened immunity due to the presence of pre-existing maternal antibodies (Feunou et al., 2016; Saso and Kampmann, 2017). Maternal immunization – a public health strategy aimed at boosting maternal-to-fetal transfer of protective antibodies – has demonstrated significant potential in providing protective immunity for the newborn prior to the infant’s ability to generate a robust immune response to vaccination. Pathogens targeted by maternal immunization strategies include respiratory pathogens that can be life-threatening to newborns, such as pertussis, and common pathogens harmful to both pregnant people and newborns, such as influenza virus (Dabrera et al., 2015; Forsyth et al., 2015; Maertens et al., 2016).

Efficacious maternal vaccines provoke a significant antigen-specific immune response in the mother that is efficiently transferred to the fetus or newborn, either transplacentally or through breast milk. Various factors can impact the success of maternal immunization strategies. Epidemiologic studies focusing on matched mother:fetus dyads have found that the extent of immunity transferred varies significantly by antigen (Palmeira et al., 2012; Fu et al., 2016), and recent data point to unique placental sieving mechanisms that populate the infant with the most functional protective antibodies in the first days of life (Jennewein et al., 2017; Jennewein et al., 2019; Atyeo et al., 2021b). Emerging data demonstrate the presence of anti-SARS-CoV-2 IgG in umbilical cord blood following maternal vaccination with mRNA vaccines, with antibody transfer ratios (i.e. ratio of maternal antibody to cord blood antibody) showing a strong correlation with both maternal antibody levels, time elapsed since vaccination, and whether one or both doses had been received (Gray et al., 2021; Mithal et al., 2021; Prabhu et al., 2021; Rottenstreich et al., 2021). In an analysis of neutralizing antibody (NAb) titers in 10 maternal:cord dyads, NAb were detectable in 8 of 10 neonates; of those newborns with undetectable NAb, one mother had not yet received the second vaccine dose, and one mother was only 7 days from the second dose at delivery (Gray et al., 2021).

Whether maternal vaccination can be tuned to shape the quantity and quality of antibodies delivered to the infant remains incompletely understood. Timing of vaccine administration during pregnancy appears to impact antibody transfer from mother to umbilical cord for vaccines routinely recommended in pregnancy, such as Tdap and the seasonal influenza hemagglutinin (HA) vaccines (Eberhardt et al., 2016; Cuningham et al., 2019). While influenza vaccine administration is timed to optimize maternal immunity relative to flu season rather than to optimize neonatal immunity *via* placental transfer, it has been noted that administration of flu vaccine in the third trimester results in greater neonatal titers than administration in the second or first trimester (Schlaudecker et al., 2018; Cuningham et al., 2019). In contrast, Tdap vaccine is recommended for administration in each pregnancy solely to optimize protection of the neonate against pertussis (ACOG, 2018). Similar to findings observed for the influenza vaccine, optimal transfer of anti-pertussis antibodies was noted with vaccination in the late second and early third trimester (Eberhardt et al., 2016; Healy et al., 2018). Importantly, vaccine-generated responses to pertussis protein and HA may differ from responses to the COVID-19 vaccines in pregnancy, as HA and pertussis protein are typically recall antigens, i.e. the host has some immunological memory due to either prior vaccination efforts or exposure, while COVID-19 vaccination in pregnancy may be eliciting a *de novo* immune response to a pathogen never before seen by the body.

Although limited by small numbers and to pregnant people primarily vaccinated in the third trimester, recent studies have demonstrated that timing of vaccination does appear to play a role in the transplacental antibody transfer of anti-SARS-CoV-2 antibody in pregnant individuals not previously infected with SARS-CoV-2 (Gray et al., 2021; Mithal et al., 2021; Rottenstreich et al., 2021). In a study of 27 vaccinated pregnant people, an average anti-SARS-CoV-2-specific antibody transfer ratio of 1.0 was identified, with a positive association identified between transfer ratio and latency from vaccination to delivery (mean latency of 6 weeks in the cohort) (Mithal et al., 2021). In a study of 20 pregnant people who received the BNT162b2 mRNA vaccine in Israel, anti-S- and anti-RBD cord blood titers directly correlated with increasing time since first mRNA vaccine dose, yet placental transfer ratios were overall low (0.3 to 0.4 for anti-RBD and anti-S-specific antibodies, respectively), likely due to shorter vaccine to delivery latency of approximately four weeks (Rottenstreich et al., 2021). In a study comparing cord:maternal transfer ratios of vaccine-elicited *vs* infection-elicited antibodies, transplacental transfer was comparable between individuals infected with SARS-CoV-2 earlier in pregnancy (15-30 weeks) and those vaccinated with the BNT162b2 mRNA vaccine in the third trimester (Beharier et al., 2021), leading the investigators to posit that anti-SARS-CoV-2 antibodies generated by natural infection may require an increased time interval to transit to cord blood compared to those generated by third trimester vaccination.

Emerging data point to the importance of the Fc-receptor (FcR) binding domain in recruiting the innate immune response in COVID-19 (Schäfer et al., 2021), as well as in selective transplacental antibody transfer of highly functional antibodies (Wilcox et al., 2017; Jennewein et al., 2019; Clements et al., 2020; Atyeo et al., 2021a). Similar to observations in pregnant people infected with SARS-CoV-2 (Atyeo et al., 2021b), in their study of pregnant/lactating individuals receiving either mRNA vaccine during pregnancy and lactation, Atyeo et al. identified that vaccination during pregnancy resulted in enrichment of highly functional RBD-specific FcGR3a binding antibodies in cord blood, despite overall lower cord anti-SARS-CoV-2-specific titers compared to maternal titers (Atyeo et al., 2021a). More comprehensive analyses of factors affecting the neutralizing capability and functionality of vaccine-induced, transplacentally-transferred antibodies (e.g. analysis beyond antibody titers alone) are warranted, to fully understand how maternal vaccination to SARS-CoV-2 might protect the newborn.

## Protection After Birth: Maternal-Infant Transfer of Vaccine-Induced Immunity in Breastmilk

It is well known that breastmilk contains protective maternal immunoglobulins that contribute to the development of the infant’s immune system (Atyeo and Alter, 2021; Rio-Aige et al., 2021). Although lactating people were excluded from vaccine trials, many received the vaccine during its initial release to the public, and were included in observational studies. In a study of 84 lactating people who received the two-dose Pfizer/BioNTech vaccine, 61.8% of individuals’ breastmilk samples tested positive for anti-SARS-CoV-2 specific IgA at 2 weeks after the prime, and 86.1% 4 weeks after the prime (1 week after the boost) (Perl et al., 2021). Levels of anti-SARS-CoV-2-specific IgG remained low for the first 3 weeks but increased at week 4, and by weeks 5 and 6, 97% of breastmilk samples tested positive (Perl et al., 2021). While this study did not assess neutralization activity *per se*, previous reports have demonstrated that anti-SARS-CoV-2 breastmilk antibodies from natural infection do have neutralizing capacity, which correlates strongly with anti-RBD IgA titers (Pace et al., 2021). Similar findings were reported in the assessment of breastmilk obtained from 31 lactating participants receiving either the Pfizer/BioNTech or Moderna mRNA vaccines, in which robust induction of anti-SARS-CoV-2-specific IgG, IgA and IgM was identified in breastmilk following the prime, with an increase in breastmilk IgG, but not IgA or IgM, following the boost (Gray et al., 2021). The duration of antibody-mediated protection provided by breastmilk-derived antibodies in newborns and infants is not expected to last much beyond the time of breastfeeding, as the mucosally-delivered IgA antibodies lack the durability of IgG antibodies in the blood (Atyeo and Alter, 2021). These data are particularly relevant in designing strategies to assist in the protection of vulnerable newborns, such as infants born preterm or those who are otherwise immunocompromised, in communities with high SARS-CoV-2 prevalence, or in households in which the ability to isolate from infected family members may be compromised.

## Future Directions

Infection with SARS-CoV-2 has the ability to generate long-lasting immunological memory in non-pregnant populations (Dan et al., 2021; Sette and Crotty, 2021), and emerging data from mRNA COVID-19 vaccines point to similar long-lasting serological protection, with demonstrable binding and neutralizing antibodies up to 8 months post-vaccination (Doria-Rose et al., 2021). Whether vaccines can generate the same long-lasting functional immunity in individuals when administered during pregnancy or lactation is not yet known, however preliminary findings described in this review suggest that pregnant and lactating individuals mount a comparably robust initial serological response to that observed in non-pregnant reproductive-age women. Continued longitudinal assessment of vaccine-induced titers in cohorts of people vaccinated during pregnancy and lactation are critical to answering these questions. Data on the safety, reactogenicity, and immunogenicity of the Janssen/Johnson & Johnson Ad26-vector vaccine in pregnant people are lacking and will be important to collect given recent concerns regarding the rare risk of cerebral venous sinus thrombosis in women less than 50. Similarly, if the NVX-CoV2373 protein-adjuvanted COVID-19 vaccine (Novavax’s Covovax) (Heath et al., 2021) is granted EUA, responses to this vaccine in pregnant and lactating individuals will be important to study. As is true in non-pregnant populations, vaccine-induced immunological protection against emerging variants, including the Delta variant, will be important to assess in individuals who received their initial vaccination series during pregnancy. Whether a “booster” dose has utility in the pregnant population, or in individuals who were vaccinated during pregnancy, will also be important to assess.

Complete pregnancy outcomes data from people vaccinated in the first and early second trimesters are not yet available as most of these pregnancies are ongoing. Durability of IgG in the blood of neonates born to vaccinated mothers has not yet been defined, nor has whether the anti-SARS-CoV-2 IgG generated influences the response to other childhood vaccines. Information on postnatal outcomes and offspring development will require long term follow-up of children born to individuals who received the vaccine during pregnancy. Challenges in vaccine uptake in pregnant people persist despite evidence of benefit and lack of evidence of harm and strong recommendations from the medical and public health communities (Mercede Sebghati, 2021), and these challenges may be even greater in minority communities and low- and middle-income countries (Kochhar et al., 2019; Webb Hooper et al., 2021). It is therefore critically important to continue to rigorously collect and responsibly report these data to provide evidence-based guidance for pregnant and lactating individuals.

## Author Contributions

LS and AE conceived of the topic of this review and drafted the manuscript. JS and PF contributed to drafting the manuscript. All authors contributed to the article and approved the submitted version.

## Funding

Eunice Kennedy Shriver National Institute of Child Health and Human Development: 3R01HD100022-02S2 to AE and K12HD103096-01 to LS. AE is also supported by the Claflin Award from Massachusetts General Hospital Executive Committee on Research.

## Conflict of Interest

The authors declare that the research was conducted in the absence of any commercial or financial relationships that could be construed as a potential conflict of interest.

## Publisher’s Note

All claims expressed in this article are solely those of the authors and do not necessarily represent those of their affiliated organizations, or those of the publisher, the editors and the reviewers. Any product that may be evaluated in this article, or claim that may be made by its manufacturer, is not guaranteed or endorsed by the publisher.
